# A methodology to extract outcomes from routine healthcare data for patients with locally advanced non-small cell lung cancer

**DOI:** 10.1186/s12913-018-3029-6

**Published:** 2018-04-11

**Authors:** Swee-Ling Wong, Kate Ricketts, Gary Royle, Matt Williams, Ruheena Mendes

**Affiliations:** 1 Department of Clinical Oncology, University College London Hospital, 235 Euston Road, London, NW1 2BU UK; 2 Division of Surgery and Interventional Science, University College London, London, UK; 3 Department of Medical Physics and Biomedical Engineering, University College London, London, UK; 4 Department of Clinical Oncology, Charing Cross Hospital, Fulham Palace Road, London, UK; 5 Computational Oncology Group, Institute for Global Health Improvement, Imperial College, London, UK

**Keywords:** LA NSCLC, Outcomes, Routine datasets

## Abstract

**Background:**

Outcomes for patients in UK with locally advanced non-small cell lung cancer (LA NSCLC) are amongst the worst in Europe. Assessing outcomes is important for analysing the effectiveness of current practice. However, data quality is inconsistent and regular large scale analysis is challenging.

This project investigates the use of routine healthcare datasets to determine progression free survival (PFS) and overall survival (OS) of patients treated with primary radical radiotherapy for LA NSCLC.

**Methods:**

All LA NSCLC patients treated with primary radical radiotherapy in a 2 year period were identified and paired manual and routine data generated for an initial pilot study. Manual data was extracted information from hospital records and considered the gold standard. Key time points were date of diagnosis, recurrence, death or last clinical encounter. Routine data was collected from various data sources including, Hospital Episode Statistics, Personal Demographic Service, chemotherapy data, and radiotherapy datasets. Relevant event dates were defined by proxy time points and refined using backdating and time interval optimization. Dataset correlations were then tested on key clinical outcome indicators to establish if routine data could be used as a reliable proxy measure for manual data.

**Results:**

Forty-three patients were identified for the pilot study. The manual data showed a median age of 67 years (range 46- 89 years) and all patients had stage IIIA/B disease. Using the manual data, the median PFS was 10.78 months (range 1.58–37.49 months) and median OS was 16.36 months (range 2.69–37.49 months). Based on routine data, using proxy measures, the estimated median PFS was 10.68 months (range 1.61–31.93 months) and estimated median OS was 15.38 months (range 2.14–33.71 months). Overall, the routine data underestimated the PFS and OS of the manual data but there was good correlation with a Pearson correlation coefficient of 0.94 for PFS and 0.97 for OS.

**Conclusions:**

This is a novel approach to use routine datasets to determine outcome indicators in patients with LA NSCLC that will be a surrogate to analysing manual data. The ability to enable efficient and large scale analysis of current lung cancer strategies has a huge potential impact on the healthcare system.

**Electronic supplementary material:**

The online version of this article (10.1186/s12913-018-3029-6) contains supplementary material, which is available to authorized users.

## Background

Lung cancer is a leading cause of cancer-related mortality world-wide with approximately 70% of patients presenting with locally advanced or metastatic disease [1]. In the UK, outcomes for patients with LA NSCLC are amongst the worst in Europe and local recurrence occurs in up to 50% of patients despite improvements in 5 year survival [2, 3]. This has highlighted a need to not only identify causes of this deficit and advance treatment strategies, but a need for frequent large scale analysis of outcomes to assess the effectiveness of such treatments.

Radiotherapy plays an important role in the treatment of patients with locally advanced disease and national cancer strategies have been implemented to incentivize centres to formally assess radiotherapy outcomes with the introduction of an outcomes-based commissioning framework [4]. As a result, there is a recognised need to be able to assess, qualify and quantify the quality of radiotherapy practice which is valuable for research and strategic planning of service provision.

Progression free survival (PFS) and overall survival (OS) are key outcome measures for lung cancer that are important assessment tools of the effectiveness of an institution’s lung cancer strategy. PFS has become an increasingly important outcome measure in clinical trials, used as a surrogate for OS that is less influenced by subsequent therapies, and important for evaluating treatment response. Measuring PFS is in itself a challenge due to an inconsistency of definition and use in the literature and measurement accuracy [5, 6]. To reliably determine these outcomes measures, the quality, completeness and consistency of data recording is important as well as the ability to efficiently interpret these. Manually collected prospective data taken from patients’ notes, as collected in trials, is considered the gold standard in most accurately identifying clinically significant dates for patients’ investigation and management pathways. However, data quality can be inconsistent and collecting it is labour- intensive, making assessment of large numbers time-consuming. Routine datasets are nationally collected patient data, including hospital episodes statistic (HES), radiotherapy database (RTDS), systemic anti-cancer therapy (SACT) and personal demographics service (PDS).

Information from routinely collected electronic datasets is inexpensive and its use in population- based studies to investigate disease incidence, mortality and public health issues has long been established. There has been growing interest in using routine data to assess clinical outcomes [7], particularly in cancer management, in the hope that regular feedback will facilitate improved outcomes [8–12]. Whilst dates of diagnosis and recurrence may not be directly captured in the data it is possible to identify information to serve as surrogates for these relevant time points and Ricketts et al. recently demonstrated that routine data could be used to estimate OS and PFS in patients with head and neck cancers treated with radical radiotherapy [13, 14].

The aim of this paper is to develop and optimise a methodology to extract OS and PFS from routinely collected electronic healthcare data for patients treated with primary radical radiotherapy for LA NSCLC that will enable information to be evaluated effectively and efficiently.

## Methods

All patients with LA NSCLC, taken to be any patient with stage IIIA/B disease (Additional file 1), treated with primary radical radiotherapy in a 2 year period (August 2013 to August 2015) in University College London Hospital, a regional referral centre, were identified for this initial pilot study of 43 patients.

For each patient, paired manual and routine datasets were generated to compare OS and PFS, based on manual data, with estimated OS and PFS based on routine data.

### Manual dataset

The manual data was extracted from hospital notes which included clinic letters, multidisciplinary team (MDT) meetings, histopathology and radiology reports, and chemotherapy and radiotherapy treatment records. The relevant time points required to calculate PFS and OS were the date of diagnosis, recurrence, and death or last known appointment.

- *Manual diagnosis date:* The diagnostic biopsy date was chosen to most accurately represent the date of diagnosis as this is when histological confirmation of disease is obtained. If the biopsy date was not available (eg. if the biopsy was performed in a different hospital) then other dates were used following a hierarchy, as defined by the UK National Lung Cancer Audit, of date of: i) imaging in the form of CT (computed tomography) and PET CT (positron emissions tomography CT) ii) admission to hospital due to this malignancy iii) patient’s evaluation at an out-patient clinic relating to this malignancy and iv) referral [15] (Table 1).
  *Manual recurrence date:* The recurrence date was taken to be the date of recognized progression, recurrence, metastases, death, or last known clinical encounter (if no progression occurred). Progression, recurrence or metastatic disease was determined by dates of any investigative procedure, including radiological scans or biopsies, which first positively identified disease recurrence (Table 1).

**Table 1:** Definitions of key time points used to calculate PFS and OS for manual data and the ICD-10 (international classification of diseases) and OPCS (Office of population censuses and surveys classification of surgical operations and procedures) codes used for diagnosis and recurrence flag events from the routine data

Time points	Definitions for manual data	Definitions for routine data
Diagnosis date	In order of preference [15]: ❖ Date of first histological or cytological confirmation of malignancy. - date when specimen taken - date of receipt by pathologist - date of pathology report ❖ Date of imaging from a CT, PET scan or other form of clinical diagnosis ❖ Date of admission to hospital because of this malignancy. ❖ When evaluated at an oncology out-patient clinic only: date of first consultation at out-patient clinic because of this malignancy ❖ Date of referral	HES ❖ Date of biopsy (taken as the optimal date of diagnosis) within pre-specified time window of X weeks of treatment initiation If not available, then the earliest within a pre-specified time window of X weeks of treatment initiation: HES ❖ First relevant ICD10 code (Additional file 4) ❖ OPCS identifying relevant time points and proxy measures for investigation (Additional file 2) and management (Additional file 3) RTDS ❖ Date of request on booking form consent date for secondary treatment. (This date must correspond to treatment that is also documented in the RTDS with “Category: Radical”) SACT ❖ Start date
Recurrence date	Any of the following that first positively identifies recurrent, progressive or metastatic disease: ❖ Date of radiological scan identifying recurrence, progressive, or metastatic disease ❖ Date of biopsy procedure confirming recurrence ❖ Date of clinic if a clinical diagnosis of recurrence, progressive, or metastatic disease is made and no scans or biopsies undertaken	The earliest within a pre-specified time window of X weeks of *secondary treatment initiation: HES ❖ ICD10 codes for secondary malignancies (Additional file 5) ❖ OPCS and ICD10 codes identifying relevant time points and proxy measures for recurrent, progressive or metastatic disease investigation (Additional file 6). RTDS ❖ Date of request on booking form consent date. (This date must correspond to treatment that is also documented in the RTDS with “Category: Palliative” If there are no secondary treatment codes (Additional file 7) but there are ICD10 codes for secondary malignancies (Additional file 5), these can be used to identify recurrence dates. If there are no ICD10 codes for secondary malignancies or investigative procedures then the start date of secondary treatment can be used: HES ❖ OPCS identifying secondary management for recurrent, progressive or metastatic disease (Additional file 7) RTDS ❖ Start date SACT ❖ Start date
Death date	❖ Date of recorded death from medical notes or clinical letters	❖ Date of recorded death on PDS
Endpoint if no recurrence or death	❖ Last known clinical encounter with any specialty (in the hospital or community) based on clinical letters or letters of correspondence from the patient or their next of kin	❖ Date of last HES, SACT, RTDS entry.

* Secondary treatment is defined as any treatment being initiated 10 weeks following completion of primary treatment, identified using relevant codes (Additional file 7)

### Routine dataset

Routine data was collected from HES, SACT, RTDS, and PDS ( Table 2).

**Table 2:** Routine datasets. This shows the national datasets available for analysis, their intended function and the patient-specific information that can be collected from the different databases

Routine dataset	Information available
PDS (Personal Demographics Service) *National electronic database and component part of the NHS Spine (the national databases of information regarding patients’ health and care)*	• Name • Address • Date of birth • NHS Number • Date of death
HES (Hospital Episodes Statistics) *Patient care data of all patients treated by the NHS in England (including private patients treated in NHS hospitals and patients resident outside England receiving treatment funded by the NHS)*	• Dates of all hospital encounters including admissions and discharge dates, outpatient appointments, and A&E attendances. • Diagnoses • Operations • Age group • Gender • Ethnicity • Area of patient’s residence
SACT (Systemic Anti-Cancer Therapy) *Clinical management information on patients undergoing chemotherapy in (or funded by) the NHS in England.*	• Demographics- including commissioner and provider initiating treatment • Clinical status-diagnosis, performance status, treatment intent • Programme and regimen- drug details, cycle and regime number, supportive medications, treatment dates • Outcome- regimen modification eg. dose reductions, cycle delays, early termination of treatment, and outcome summary.
RTDS (Radiotherapy Dataset) *Clinical management information on patients undergoing radiotherapy treatment collected locally by radiotherapy centres and submitted to the National Clinical Analysis and Specialised Applications Team.*	• Demographics- commissioner and provider initiating treatment • Clinical status- diagnosis, treatment intent, history of previous radiotherapy (diagnosis relating to that treatment, treatment intent, dose, fractionation, site treated, dates of referral and of treatment). • Dose prescription- dose and fractionation regime, treatment site • Outcome- actual dose delivered, treatment dates

Surrogates were identified as suitable proxy measures for dates of diagnosis and recurrence events (Table 1).

- *Routine diagnosis date:* This was taken to be the corresponding date of biopsy OPCS codes (Additional file 2) (available for all patients investigated within our centre), so long as it fell within a pre-specified time window of x weeks of lung cancer treatment initiation (Additional file 3) (see section “Interval definitions for back-dating”). If biopsy codes were not found on routine data, proxy time points were used as surrogates as long as they occurred within this pre-specified time window from start of primary treatment: the earliest of (i) the first relevant ICD10 codes denoting lung malignancy (Additional file 4) or (ii) other investigative OPCS codes (Additional file 2).
- *Routine recurrence date:* The date of progression, recurrence or metastases was identified using (i) ICD 10 codes for secondary malignancies (Additional file 5) or investigative procedures (Additional file 6) which occurred within a pre-specified time window prior to secondary treatment (Additional file 7) initiation (ii) ICD10 codes identifying secondary malignancies if no secondary treatment codes appeared (iii) the start date of secondary treatment if no ICD10 codes for secondary malignancies or investigative procedures were seen on routine data.

Secondary treatment was defined as any treatment event occurring more than 10 weeks after the end of primary treatment (last day of radiotherapy or chemotherapy, whichever occurred last) and further identified by OPCS codes in Additional file 7.

### Survival intervals

PFS was taken to be the time interval between the diagnosis date and the date of progression, recurrence or metastases. If no progression occurred the date of last known clinical encounter or death was used.

OS was taken to be the time interval between the diagnosis date to the date of death from any cause or date of last known clinical encounter (if the patient was still alive at the time of analysis).

Key code tables were generated to aid interpretation of the routine data (Additional files 2, 3, 4, 5, 6 and 7), enabling the identification of codes signifying the relevant time points. The datasets were analysed separately in this manner and then merged to create a timeline.

### Code identification and classification

Codes were identified and sorted according to diagnostic ICD 10 codes consistent with lung malignancy (Additional files 4 and 5) and OPCS codes consistent with diagnostic investigations (including biopsies and CT or CT PET imaging) (Additional files 2 and 6) and management strategies (Additional files 3 and 7), separated into radiotherapy, chemotherapy and interventional treatment.

- ICD-10 codes indicating primary site lung malignancies (Additional file 4)

All codes relating to “malignant neoplasm of bronchus or lung” [C34], “malignant neoplasm of heart, mediastinum and pleura” [C38], and “Secondary and unspecified malignant neoplasm of intrathoracic lymph nodes” [C77.1] were identified as the majority of patients with LA NSCLC have mediastinal lymph node involvement. The additional code, “Abnormal findings on diagnostic imaging of lung” [R91] was included given the first suspicion of lung malignancy arises from abnormalities seen on chest x-rays or CTs, acknowledging this does not confirm diagnosis.

b. OPCS codes identified for primary diagnostic event (Additional file 2)

Codes identified as surrogates for the diagnosis of LA NSCLC included biopsies of the lung, pleura and mediastinal lymph nodes and procedures whereby specimens are obtained for cytological confirmation of malignancy. Imaging with body and head CT and PET CT are important for staging of disease and glomerular filtration rate testing is standardly performed for any patient being considered for chemotherapy.

c. OPCS codes identifying primary management (Additional file 3)

Primary management codes included those denoting treatment with radical radiotherapy (identified as intensity modulated radiotherapy [X67.1] and complex conformal radiotherapy [X67.7]) and chemotherapy. Interventional codes included endovascular stent placement [L76.9] and insertion of stent into vena cava [L79.3] (which means the patient experienced superior vena cava obstruction secondary to a locally advanced tumour in the lung apex) and required treatment with stent insertion.

d. ICD-10 codes indicating secondary site malignancies or complications from recurrent/ progressive/ metastatic disease (Additional file 5)

The codes identified for the diagnosis of recurrent, progressive or metastatic disease mostly included those with “Secondary & unspecified malignant neoplasm of-” as this implies that malignant disease has metastasized to this site; and codes that identified complications from metastases, such as cerebral oedema [G93.6], which can result from cerebral metastases. Additionally, there were codes that overlapped with those identifying primary presentation as recurrent and metastatic disease can present with similar complications depending on the location of disease.

e. OPCS codes identified for recurrent, progressive or metastatic disease diagnostic event (Additional file 6)

Codes identified as surrogates for the diagnosis of recurrent, progressive or metastatic disease overlapped codes for primary presentation, as biopsies are used to confirm recurrence and imaging is used to re-stage disease. Additional imaging OPCS codes included those denoting MRI spines ([U211 AND Z06.1], [U21.1 AND Z06.2], [U21.1 AND Z99.2], [U21.1 AND Z06.3]) and bone scans [U14.1], as these are not routinely done at initial staging but are performed to investigate metastases to the spine and bones, respectively.

f. OPCS codes identifying secondary management for recurrent, progressive or metastatic disease (Additional file 7)

Radiotherapy OPCS codes for “simple radiotherapy” ([X67.5], [Y91.2]) were used as they indicate that treatment is non- curative (as opposed to “complex radiotherapy” [X67.7], which indicates treatment is radical with the intention of cure). The only exception to this rule is that “Preparation for intensity modulated radiation therapy” [X67.1] (considered complex radiotherapy that is usually delivered in the radical setting) is also used to code for SABR (stereotactic ablative radiotherapy), which can be used to treat oligometastatic (single or few systemic metastases that are amenable to surgery or ablative therapy) disease.

For chemotherapy OPCS codes, only “Delivery of exclusively oral chemotherapy for neoplasm” [X73.1] is exclusive to patients being treated for recurrent or metastatic disease because there are no oral chemotherapy drugs currently used in the radical setting. The SACT data can be used in conjunction with the OPCS codes as it details the specific chemotherapy drugs delivered to patients and this information can be used to help discriminate curative or non-curative (palliative) treatment as some drug regimens are used exclusively as palliative treatment.

Interventional codes for endovascular stent placement and insertion of stent into vena cava were also included here as superior vena cava obstruction can be a complication of locally recurrent or metastatic disease requiring treatment with stent insertion.

### Interval definitions for back-dating

**Fig. 1:**
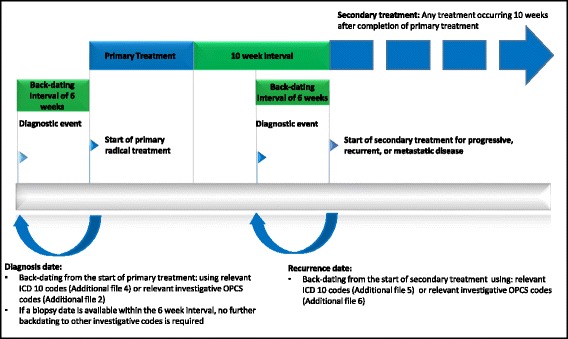
Schematic showing back-dating intervals used for optimization of key time points extracted from routine data. The date of biopsy is taken to be the date of diagnosis, so long as this date is within a 6 week period of an OPCS code indicating the start of primary treatment. If there is no biopsy date, then any diagnostic event or relevant ICD 10 code (whichever occurs first) occurring within a 6 week period prior to an OPCS code indicating the start of primary treatment is taken to be the date of diagnosis of primary disease. For example, an OPCS code for investigative imaging occurring within 6 weeks prior to treatment, implies there was already clinical suspicion of malignancy at the time of that scan. For the date of recurrence, progressive or metastatic disease, any diagnostic event or ICD10 code (whichever occurs first) occurring within a 6 week period prior to an OPCS code indicating the start of secondary treatment is taken to be the date of recurrence. Any treatment event occurring after 10 weeks after completion of primary treatment was interpreted as secondary treatment. If no secondary treatment has been given then a secondary malignancy ICD 10 code (Additional file 5) can be used to identify recurrent disease

The process of interval back-dating was used to optimize the correlation of manual and routine intervals when using proxy time points from the routine data and filter out diagnostic events that yielded negative results (Fig. 1). For the date of diagnosis, a back-dating window of 6 weeks (1.5 months) was chosen as the interval during which a diagnostic event might occur prior to the initiation of primary treatment (denoted by a relevant investigative OPCS code), or ICD10 code indicating primary diagnosis (whichever occurred earliest). If a biopsy OPCS code was available, this was taken to be the date of diagnosis, so long as it occurred within 6 weeks prior to the start of primary treatment, with no further back-dating to other investigate codes. The same backdating interval was used to identify the diagnostic events for secondary malignancy presentation prior to initiation of secondary treatment.

Correlation of these datasets were then tested on the key clinical outcome indicators of OS and PFS to establish if routine data could be used as a reliable proxy measure for manual data.

## Results

### Patients’ characteristics

We identified 43 consecutive patients for this pilot study, 27 of whom were men and 16 women. Patient demographics are displayed in Table 3. The median age was 67 years (range 46- 89 years) and all patients had stage IIIA/B disease. The majority of patients were PS (performance status) 0–1 but for 6/43 patients the PS was not recorded and 3/43 patients had a PS of 2. 20/43 patients had the optimal cCRT (concurrent chemoradiotherapy) [16], 9/43 patients had sCRT (sequential chemoradiotherapy), 13/43 patients had radical radiotherapy alone, and 1 patient unconventionally received gefitinib followed by radical radiotherapy (Table 3).

**Table 3:** Patients’ characteristics. NR (Not recorded). PS (Performance status)* (Additional file 8 [21]), EGFR (epidermal growth factor receptor), EGFR mutation (epidermal growth factor receptor with a sensitizing mutation to targeted therapy), ALK (anaplastic lymphoma kinase), Kras (K-rat sarcoma), WT (wild type) meaning no sensitizing mutations are found. cCRT (concurrent chemoradiotherapy), sCRT (sequential chemoradiotherapy). RT (radiotherapy). 4 cycles of chemotherapy are usually given. CV (cisplatin and vinorelbine), CarboV (carboplatin and vinorelbine), GCis (gemcitabine and cisplatin), GCarb (gemcitabine and carboplatin), Pemcarbo (pemetrexed and carboplatin), CisN (cisplatin and navelbine). AE (adverse event)

Patient	Age range	PS	Stage	Histology	Treatment
1	45-49y	PS1	IIIA	Squamous cell carcinoma	cCRT (CVx4; 64Gy in 32#)
2	65-69y	PS0	IIIA	Squamous cell carcinoma	sCRT (GCarb × 4; 64Gy in 32#)
3	70-74y	PS1	IIIA	Squamous cell carcinoma	cCRT (CV ×1-stopped due to AE; 64Gy in 32#)
4	70-74y	PS1	IIIA	Squamous cell carcinoma	sCRT (GCis ×4; 55 Gy in 20#)
5	80-84y	PS1	IIIA	Adenocarcinoma. EGFR WT	sCRT (pemcarbo ×2- stopped due to AE; 64Gy in 32#)
6	55-59y	PS1	IIIA	Adenocarcinoma EGFR WT	cCRT (CV ×4, 64Gy in 32#)
7	70-74y	PS1	IIIB	Squamous cell carcinoma	sCRT (GCarbo ×3- stopped due to AE; 55Gy in 20#)
8	65-69y	PS0	IIIA	Squamous cell carcinoma	cCRT (CVx3; 55gy in 20#)
9	65-69y	NR	IIIA	Squamous cell carcinoma	RT alone: 55gy in 20#
10	55-59y	NR	IIIA	Squamous cell carcinoma	RT alone: 64Gy in 32#
11	65-69y	NR	IIIB	Squamous cell carcinoma	sCRT (GCisx2 switched to GCarbo x 2 due to AE; 64Gy in 32#
12	65-69y	NR	IIIA	Adenocarcinoma. EGFR and ALK WT	cCRT (CV ×4, 64Gy in 32#)
13	75-79y	PS1	IIIA	Adenocarcinoma. EGFR mutation	Gefitinib ×6 followed by 55 in 20#
14	65-69y	PS0	IIIA	Adenocarcinoma. EGFR and ALK WT	cCRT (CVx1 switched to CarboV ×3 due to AE; 64Gy in 32#)
15	65-69y	PS1	IIIA	High grade dysplasia at least; no definitive invasive malignancy	cCRT (CV ×4; 64Gy in 32#)
16	55-59y	PS1	IIIA	Squamous cell carcinoma	cCRT (CV ×2; 64Gy in 32#)
17	70-74y	NR	IIIB	Adenocarcinoma. EGFR and ALK WT	RT alone: 64Gy in 32#
18	75-79y	PS0	IIIB	Adenocarcinoma. EGFR and ALK WT	cCRT (CisN; 64Gyin 32#)
19	80-84y	PS2	IIIB	Squamous cell carcinoma	RT alone: 64Gy in 32#
20	50-54y	PS1	IIIA	Adenocarcinoma. EGFR and ALK WT	cCRT (CV ×4; 64Gy in 32#)
21	50-54y	PS0	IIIA	Adenocarcinoma. EGFR and ALK WT	cCRT (CV ×3; 64Gy in 32#)
22	55-59y	PS1	IIIB	Adenocarcinoma. EGFR and ALK WT	RT alone: 64Gy in 32#
23	70-74y	PS1	IIIA	Squamous cell carcinoma	RT alone: 55Gy in 20#
24	75-79y	PS1	IIIA	Adenocarcinoma. EGFR and ALK WT	sCRT (CVx4; 64Gy in 32#)
25	80-84y	PS1	IIIA	PD carcinoma(no comment on EGFR/ALK)	RT alone: 55Gy in 20#
26	60-64y	PS0	IIIA	Adenocarcinoma. EGFR and ALK WT	sCRT (CV ×2 switched to CarboV ×2 due to AE; 64Gy in 32#)
27	80-84y	PS0	IIIA	Squamous cell carcinoma	cCRT (CV ×4; 64Gy in 32#)
28	45-49y	PS1	IIIB	Squamous cell carcinoma	cCRT (CV ×4; 64Gy in 32#)
29	65-69y	PS1	IIIA	Squamous cell carcinoma	cCRT (CarboVx3; 64Gy in 32#)
30	45-49y	PS1	IIIA	Adenocarcinoma-insufficient material for ALK/EGFR testing	sCRT (cispem ×4; 64Gy in 32#)
31	65-69y	PS0	IIIA	Adenocarcinoma. EGFR and ALK WT	cCRT (CV ×4; 64Gy in 32#)
32	60-64y	PS1	IIIA	Adenocarcinoma. EGFR and ALK WT	sCRT (cispemx2 switched to CV ×2 due to AE; 64Gy in 32#)
33	60-64y	PS1	IIIB	Squamous cell carcinoma	cCRT (CV ×4; 64Gy in 32#)
34	70-74y	PS1	IIIB	Squamous cell carcinoma	RT alone: 64Gy in 32#
35	60-64y	PS1	IIIB	Adenocarcinoma. EGFR and ALK WT, KRAS mutation	cCRT (CV ×4; 64Gy in 32#)
36	45-49y	PS1	IIIA	NSCLC-not possible to further differentiate tumour type	cCRT (CV ×4; 64Gy in 32#)
37	70-74y	PS2	IIIA	Squamous cell carcinoma	RT alone: 55Gy in 20#
38	65-69y	PS1	IIIB	Adenocarcinoma. EGFR and ALK WT	cCRT (CV ×4; 64Gy in 32#)
39	55-59y	PS1	IIIB	Adenocarcinoma. EGFR and ALK WT	cCRT (CV ×6; 64Gy in 32#)
40	80-84y	PS1	IIIA	Adenocarcinoma	RT alone: 55Gy in 20#
41	75-79y	NR	IIIB	Adenocarcinoma. EGFR and ALK WT	RT alone: 64Gy in 32#; declined chemotherapy
42	60-64y	PS2	IIIA	Adenocarcinoma. EGFR and ALK WT	RT alone: 55Gy in 20#
43	85-89y	PS1	IIIA	Squamous cell carcinoma	RT alone: 55Gy in 20#

Twenty two patients had adenocarcinomas (18 of whom had no sensitizing mutations and 3 with unknown EGFR/ALK status), 19 had squamous cell carcinomas, in 1 patient it was not possible to further differentiate the tumour beyond determining that it was a NSCLC.1 patient had no definitive invasive malignancy demonstrated on biopsy but was treated due to high clinical suspicion.

### Survival and recurrence

**Fig. 2:**
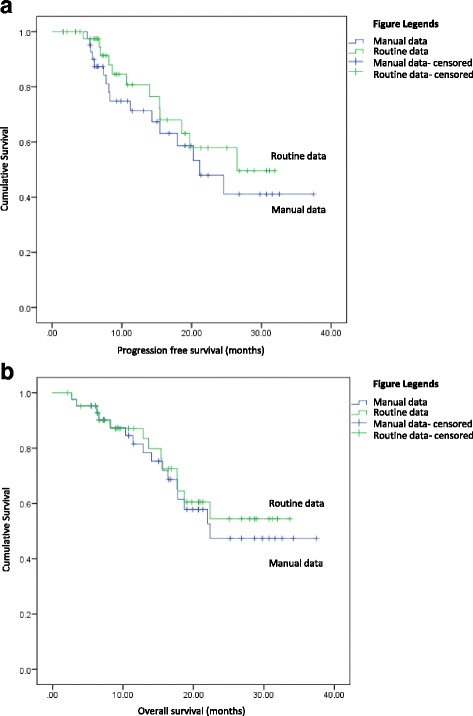
**a** Kaplan Meier Curve for PFS (in months). Survival curves for the routine (green line) and manual (blue line) data are shown. 27/43 events censored from the manual data and 31/43 events censored from the routine data. Wilcoxon signed-ranks test statistic 1.10, p(0.29). **b** Kaplan Meier Curve for OS (in months). Survival curves for the routine (green line) and manual (blue line) data are shown. 27/43 events censored from the manual data and 30/43 events censored from the routine data. Wilcoxon signed-ranks test statistic 0.08, p(0.78)

Using the manual data, the median PFS was 10.78 months (range 1.58–37.49 months) and median OS was 16.36 months (range 2.69–37.49 months). Based on the routine data, using proxy measures, the median PFS was estimated at 10.68 months (range 1.61–31.93 months) and median OS was estimated at 15.38 months (range 2.14–33.71 months) (Fig. 2a and b).

The routine methodology failed to detect 4 recurrences and 3 deaths resulting in increased censoring of events and a separation of the curves that was not statistically significant for either endpoint.

### Data correlation

**Fig. 3:**
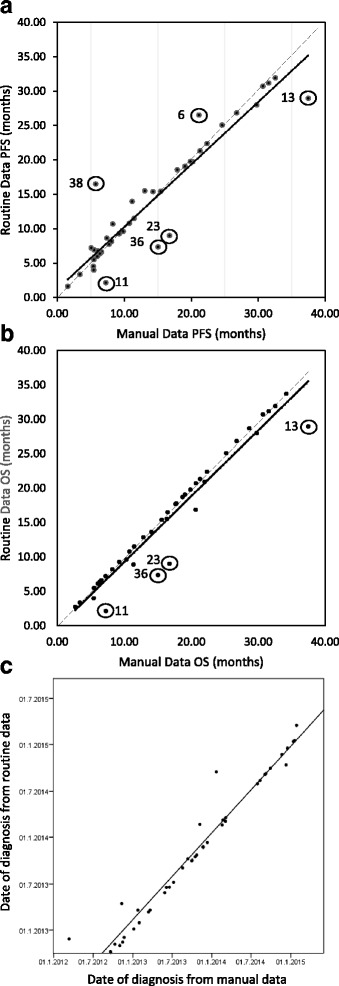
**a**. Correlation between manual and routine derived PFS intervals. Correlation coefficient of 0.94, *p* < 0.0001. Solid line represents the line of best fit for the data points. Dashed line represents the correlation line if the manual and routine data were equal. Outliers are circled and identified with their patient number corresponding to Table 3. **b** Correlation between manual and routine derived OS intervals. Correlation coefficient of 0.97, *p* < 0.0001. Solid line represents the line of best fit for the data points. Dashed line represents the correlation line if the manual and routine data were equal. Outliers are circled and identified with their patient number corresponding to Table 3. **c** Correlation between manual and routine dates of diagnosis**.** Correlation coefficient of 0.98, *p* < 0.0001. Solid line represents the line of best fit for the data points

Overall the routine data underestimated the PFS (manual (mean = 13.88 months, SD = 9.31); routine (mean = 13.79 months, SD = 8.95) and OS (manual (mean = 16.49 months, SD = 9.33); routine (mean = 15.48 months, SD = 9.17) of the manual data. A paired sample t-test for the mean PFS showed a difference of 0.09 months (*p* = 0.86; 95% confidence interval − 0.86- 1.03) and 1.02 months (*p* = 0.00; 95% confidence interval 0.34–1.69) for the difference in the mean OS. However, there was good overall correlation of 0.94 (*p* = 0.00, 95% confidence interval 0.90–0.97) for PFS (Fig. 3a) and 0.97 (*p* = 0.00, 95% confidence interval 0.95–0.98) for OS (Fig. 3b).

The routine methodology correctly identified 32/43 routine diagnosis dates to within 2 weeks accuracy of the manual diagnosis dates, and of those, 21/43 dates matched exactly. 5/43 routine diagnosis dates were earlier than the manual dates (ranging from 1 to 6 days earlier). 5/43 routine diagnosis dates were outside of 2 weeks but within 4 weeks of the manual data; and for 6/43 patients, there was a > 28 day difference in routine and manual diagnosis dates, with the routine dates occurring later than the manual. 3 patients had a difference in diagnosis dates of > 100 days. Whilst the paired sample t test showed that routine data tend to suggest later diagnosis dates compared to that identified by manual data (*t* = − 2.45; *p* = 0.02) and the overall correlation was 0.98 (*p* = 0.00, 95% confidence interval 0.96–0.99) (Fig. 3c).

The sensitivity and specificity of using routine data instead of manual data to determine recurrences was 0.75 and 1, respectively. 12/16 recurrences were correctly detected when assessing the routine data alone. 4/16 routine recurrence dates were within 2 weeks of the manual diagnosis dates, and of those, 3/16 dates matched exactly. 6/16 routine diagnosis dates were outside of 4 weeks but less than 100 days of the manual data. For 2/16 patients, there was a > 100 day difference in routine and manual diagnosis dates (Fig. 3a).

The sensitivity and specificity of using routine data instead of manual data to determine death event was 0.81 and 1, respectively. 13/16 death events were correctly detected on the routine data and of those, 12/13 dates of death matched exactly and for the remaining other patient, the routine date fell within 1 week of the manual death date. For 27 patients who were still alive at the time of assessment and for whom the last clinical encounter was used as the end- interval, the manual and routine dates matched exactly for 24/27 patients (Fig. 3b).

For patients 13 and 11, diagnosis and chemotherapy (as part of sCRT) were initiated in other hospitals and followed-up continued there, resulting in missing clinical episodes on routine data but detection on manual data (as clinical correspondence letters were available). The result was later routine diagnosis dates and shorter overall routine PFS and OS. Patient 36 similarly continued follow-up in another hospital. For patients 23 and 6 a late routine diagnosis date resulted from positive diagnostic investigations falling outside the 6 week back-dating interval from treatment and alternative surrogates being used, resulting in a shorter PFS and OS. For patient 38, the routine PFS was shorter as recurrence was not detected on routine data due to individualised treatment which was not listed as a standard treatment code.

## Discussion

In this pilot study, we analyzed the PFS and OS for 43 patients with LA NSCLC treated in our regional referral centre in north London over a 2 year period. The results suggest that routine data can potentially be used to reliably estimate survival outcomes for patients with LA NSCLC treated with primary radical radiotherapy. This method relies on identifying relevant ICD-10 and OPCS codes that are used as surrogates for diagnosis and recurrence dates followed by a refining process that involves back-dating interval optimization to improve correlation.

There are some crucial considerations in defining the key time points both for the manual data and routine data interpretation: 1. Manual diagnosis dates: These followed a hierarchy with imaging following the preferred diagnostic biopsy date due to certain limitations: Whilst imaging can give a strong indication of malignancy, patients with lung cancer often have background lung disease that makes them prone to recurrent chest infections. Radiological changes seen during active chest infections make identifying malignancy less reliable. This is in contrast to identifying recurrence and/or metastatic disease when malignancy is already known, and diagnosis is often done radiologically without repeat biopsies, unless diagnosis is uncertain. 2. Manual and routine recurrence interval dates: Taken to be the date of progression, recurrence, metastases, death, or last known clinical encounter (if no progression occurred) for practical reasons- so that events would be reached. 3. Identifying secondary treatment in routine data: Any therapy starting after a 10 week interval from the last day of radiotherapy or chemotherapy (whichever was completed last) was chosen as an indicator of secondary treatment because it is standard practice for patients to have reassessment imaging at 8–12 weeks following completion of treatment. At this point, progressive or metastatic disease can be observed so a 10 week interval was selected as a compromise- too short an interval might pick up delayed primary treatment events, and too long an interval might miss the start of secondary treatment.

OS and PFS values derived from our routine data methodology correlated well with that derived from the gold standard manual data with the Wilcoxon signed-rank test results suggesting no statistically significant difference between the survival curves when assessed by manual versus routine data. Based on the manual data, the median PFS and OS was 10.78 months and 16.36 months, respectively*.* Using the proxy measures from the routine data, the estimated median PFS and OS was 10.68 months and 15.38 months, respectively. The paired sample t-tests showed the difference in the mean PFS to be small and non-significant but the difference in the mean OS to be larger and significant. However, these results correlated well overall with the manual data, giving a statistically significant correlation coefficient of 0.94 for PFS and 0.97 for OS. The high sensitivity and specificity of our method indicate that analyzing routine data does not tend to falsely identify recurrence or death events so survival estimates are less likely to be underestimated.

All discrepancies between the manual and routine data sets could be attributed to 1.Missing or inaccurately entered OPCS or ICD10 codes due to a) patients having diagnosis and/or recurrence detected with treatment initiation in other hospitals b) codes appearing on patients’ admission dates rather than the dates of the procedures themselves or c. treatment of recurrence being non-standard (eg. oligometastatic disease being treated surgically) and 2. Delays in initiation of treatment beyond the NHS England target of 31 days due to, for example, patients becoming unwell, resulting in alternative surrogates having to be used for dates of diagnosis or recurrence. The reasons for deaths escaping detection on routine data were mostly unclear although 1 patient died abroad, a situation that is perhaps less reliably updated on to the system. However, there is a time lag between the occurrence of death and its record being updated on the system, and it is possible that this affected the ability to detect death events on routine data. These all led to late diagnosis dates, late or absent recurrence dates, and/or absent death dates and subsequently inaccurately calculated PFS and OS. Although this resulted in an increased censoring of events and a separation of the survival curves, the differences were not statistically significant (Fig. 2a and b).

Our back-dating strategy, used to optimize correlation between manual and routine primary diagnosis and recurrence event dates, utilized time intervals tailored to reflect clinical practice and the clinical target times set out by NHS England (2013). This framework recommends that the maximum time from diagnosis to first definitive treatment is 1 month (or 31 days); and that for all subsequent treatments for new cases or primary and recurrent cancer, the maximum time interval is 1 month (or 31 days). Therefore, ICD10 codes consistent with a primary diagnosis or recurrent, progressive or metastatic disease, are likely to be preceded by diagnostic investigation codes within a period of up to 31 days. A longer interval of 6 weeks was chosen to avoid potentially missing relevant investigative and diagnostics flag for patients who may have started treatment beyond the 31 day target. This meant we still captured patients who may have had delays in starting treatment due to 1.patients’ choice 2.becoming unwell 3.radiotherapy re-planning requirements resulting from significant changes in anatomy or 4.an inability to start in the preferred time period due to patient load exceeding treatment capacity at that time.

The completeness of recorded information is a fundamental limitation of both manual and routine data. Manual data not only most reliably determines outcome measures but contains important details such as histological subtype, mutation status, lung function, detailed smoking status (ex-smoker, recent ex-smoker, and quantification by pack-years), response to treatment demonstrated on CT (stable disease, partial response, progressive disease), and grading of side effects from treatment. At present such information can only be identified in manual data as these are not coded in routine data. However, clinical outcome measures can be inferred or used as proxy indicators. For example, it would be reasonable to assume that ICD10 codes for oesophagitis or neutropenia in a patient receiving chemotherapy and radiotherapy might be experiencing these side effects as a direct result from their treatment. The caveat is that there may be confounding factors or comorbidities causing these problems, the severity of these side effects are not coded, and the absence of these ICD-10 codes does not mean they were not experienced. Adverse effects from treatment have an important impact on patients’ ability to complete treatment and their quality of life.

Additional limitations include potentially confusing routine information for patients who have other synchronous or metachronous malignancies (eg. head and neck and bladder cancers) where recurrences and treatment may occur. For these patients with dual pathology, where “Secondary & unspecified malignant neoplasm of-” or “Secondary malignancy of-” codes appear in the HES data, referring to the RTDS and SACT data can help distinguish if treatment is being initiated for disease relating to the lung cancer or to the other malignancy as 1) the RTDS data will state the site being treated and the relevant ICD-10 diagnosis code relating to that treatment (eg. Pelvic metastases from a lung cancer primary will have “pelvis” documented as the treatment site and an ICD10 code denoting a lung cancer primary) 2) the SACT data would inform us as to what chemotherapy is being delivered (which, in itself, might be indicative of the primary, if the regime is exclusive to lung cancer) and the primary diagnosis relating to that chemotherapy regime.

Interestingly, although it is well recognized that PS impacts OS [2, 15, 17], is used to help determine the most appropriate management course [18], and is required to be recorded in manual and routine databases, this appears to be poorly recorded in both. This perhaps reflects a view that the usefulness of a PS score is limited by the degree of subjectivity and inter-observer variability in assessment [17, 19].

There has been a recognized need to improve the quality of routine data in order to broaden its clinical application. An example of one such database developed for quality improvement is the Cancer Outcomes and Service Dataset (COSD) that has recently been introduced as the new national standard for reporting cancer in the NHS in England, having replaced the National Cancer Dataset. This system will enable the clinical details and outcomes from multidisciplinary team meetings (where all patients diagnosed with and being considered for cancer treatment are discussed) to be entered in to COSD. This has begun to be in use in our hospital and one of the changes this will have on outcomes analysis will be to ascertain a more accurate diagnosis date.

In addition, national cancer strategies [4, 20] have placed increasing emphasis on recording of clinical outcome measures to help monitor if national targets are being met which will drive the enrichment of the available clinical databases, and focus more attention on developing methods to analyse routine datasets. This will not only promote the clinical usefulness of routine data for survival outcomes but potentially for treatment toxicity and patient-screening for entry into trials.

Future work includes integrating new national datasets and testing our method on a larger cohort to see if accuracy can be improved. Whilst the identified event flags used as proxy measures and the chosen back-dating intervals reflect our local practice, we have deliberately ensured they are not specific to it such that this method is transferable to other centres. As the management of NSCLC in the UK is standardized by NICE guidelines any nuances in practice across the country are unlikely to limit the application of this technique although adjustments for optimization may be required. Once this technique has been sufficiently refined, a computational algorithm will be developed to automate this process such that large scale routine data can be processed more efficiently.

## Conclusions

This is a novel approach that uses routine datasets to determine outcome indicators in patients with LA NSCLC that has the potential to be a reliable surrogate to analyse manual data, having demonstrated a Pearson correlation coefficient of 0.94 for PFS, and 0.97 for OS. An algorithm is being developed to enable automated interpretation of routine datasets for patients with LA NSCLC and is being refined to improve data correlation. The clinical application of automated routine data interpretation goes beyond assessing survival data in LA NSCLC, and can be tailored to auto-analyse outcomes for other stages of NSCLC and/or other tumour types. The ability to enable efficient and large scale analysis of current lung cancer strategies has a huge potential impact on the healthcare system.

## Additional files

Additional file 1: Lung Cancer Staging (AJCC 7th Ed): Table describing the TNM lung cancer staging. (DOCX 18 kb)

Additional file 2: OPCS codes identified for primary presentation and investigation: Table listing biopsy OPCS codes, other diagnostic procedure OPCS codes, diagnostic imaging OPCS codes and RTDS indicators. (DOCX 18 kb)

Additional file 3: OPCS codes identifying primary management: Table listing radiotherapy OPCS codes, chemotherapy OPCS codes and interventional OPCS codes. (DOCX 17 kb)

Additional file 4: ICD-10 codes indicating primary site lung malignancies: Table listing ICD-10 codes indicating primary site lung malignancy. (DOCX 17 kb)

Additional file 5: ICD-10 codes indicating secondary site malignancies or complications from recurrent/ progressive/ metastatic disease: Table listing ICD-10 codes indicating secondary site malignancies and ICD-10 codes indicating complications from disease. (DOCX 18 kb)

Additional file 6: OPCS codes identified for recurrent, progressive or metastatic disease presentation and investigation: Table listing diagnostic procedure OPCS codes, diagnostic imaging OPCS codes and RTDS indicators. (DOCX 19 kb)

Additional file 7: OPCS codes identifying secondary management: Table listing radiotherapy OPCS codes, chemotherapy OPCS codes and interventional OPC codes. (DOCX 18 kb)

Additional file 8: ECOG/ WHO Performance Status: Table describing ECOG/ WHO Performance status grades used to reflecting patients’ fitness. (DOCX 15 kb)

## Abbreviations

AE: Adverse event
ALK: Anaplastic lymphoma kinase
cCRT: Concurrent chemoradiotherapy
COSD: Cancer outcomes and service dataset
CT: Computed tomography
ECOG: Eastern Cooperative Oncology Group
EGFR: Epidermal growth factor receptor
HES: Hospital episodes statistic
ICD 10: International classification of diseases
LA NSCLC: Locally advanced non-small cell lung cancer
MDT: Multidisciplinary team
OPCS: Office of population censuses and surveys classification of surgical operations and procedures – a Classification of Interventions and Procedures
PDS: Personal demographics service
PET CT: Positron emissions tomography CT
PS: Performance status
RT: Radiotherapy
RTDS: Radiotherapy database system
SABR: Stereotactic ablative radiotherapy
SACT: Systemic anti-cancer therapy
sCRT: Sequential chemoradiotherapy
