# Supplementary material for: A methodology to extract outcomes from routine healthcare data for patients with locally advanced non-small cell lung cancer
Source: BMC Health Serv Res. 2018 Apr 11;18:278. doi: 10.1186/s12913-018-3029-6 (PMC5896093; doi:10.1186/s12913-018-3029-6)
Supplement: Supplementary file 1 — Lung Cancer Staging (AJCC 7th Ed): Table describing the TNM lung cancer staging. (DOCX 18 kb) [file 12913_2018_3029_MOESM1_ESM.docx]

**Additional file 1. Lung Cancer Staging (AJCC 7^th^ Ed)**

| **TNM definitions** | |
| --- | --- |
| **Tis** | Carcinoma in situ |
| **T1** | Tumour <3cm, surrounded by lung or visceral pleura, without bronchoscopic evidence of invasion more proximal that the lobar bronchus |
| **T2** | Tumour more than 3cm but 7cm or less or tumour with any of the following features: involves main bronchus, 2cm or more distal to the carina, invades visceral pleura; associated with atelectasis or obstructive pneumonitis that extends to the hilar region but does not involve the entire lung |
| **T3** | Tumour more than 7cm or one that directly invades any of the following: parietal pleura, chest wall (including superior sulcus tumours), diaphragm, phrenic nerve, mediastinal pleural, parietal pericardium; or tumour in the main bronchus less than 2cm distal to the carina but without involvement of the carina; or associated atelectasis or obstructive pneumonitis of the entire lung or separate tumour nodules(s) in the same lobe. |
| **T4** | Tumour of an size that invades any of the following: mediastinum, heart, great vessels, trachea, recurrent laryngeal nerve, oesophagus, vertebral body, carina, separate tumour nodules (s) in a different ipsilateral lobe |
| **N0** | **No regional lymph node metastases** |
| **N1** | Metastasis in ipsilateral peribronchial and/or ipsilateral hilar lymph nodes and intrapulmonary nodes, including involvement by direct extension |
| **N2** | Metastasis in ipsilateral mediastinal and/or subcarinal lymph node(s) |
| **N3** | Metastasis in contralateral mediastinal, contralateral hilar, ipsilateral or contralateral scalene, or supraclavicular lymph node(s) |
| **M0** | No distant metastasis |
| **M1a** | Separate tumour nodules(s) in a contralateral lobe, tumour with pleural nodules or malignant pleural (or pericardial) effusion |
| **M1b** | Distant metastasis (in extrathoracic organs) |
| **Stage** | |
| **IA** | T1a-1bN0M0 |
| **IB** | T2aN0M0 |
| **IIA** | T1a-T2aN1M0; T2bN0M0 |
| **IIB** | T2bN1M0; T3N0M0 |
| **IIIA** | T3N1M0; T1a-3N2M0; T4N0M0 |
| **IIIB** | T4N2M0; T1a-4N3M0 |
| **IV** | Any T, any N, M1a-b |
