# Supplementary material for: A methodology to extract outcomes from routine healthcare data for patients with locally advanced non-small cell lung cancer
Source: BMC Health Serv Res. 2018 Apr 11;18:278. doi: 10.1186/s12913-018-3029-6 (PMC5896093; doi:10.1186/s12913-018-3029-6)
Supplement: Supplementary file 2 — OPCS codes identified for primary presentation and investigation: Table listing biopsy OPCS codes, other diagnostic procedure OPCS codes, diagnostic imaging OPCS codes and RTDS indicators. (DOCX 18 kb) [file 12913_2018_3029_MOESM2_ESM.docx]

**Additional file 2. OPCS codes identified for primary presentation and investigation.**

| **Biopsy OPCS Codes** | |
| --- | --- |
| E59.1 | Needle biopsy of lesion of lung |
| E59.3 | Biopsy of lesion of lung NEC |
| T09.2 | Open biopsy of lesion of pleura |
| T12.1 | Drainage of lesion of pleura NEC |
| T12.3 | Aspiration of pleural cavity |
| T87.4 | Excision or biopsy of mediastinal lymph node |
| Y20.4 | Fine needle aspiration NOC |
| Y21.1 | Brush cytology of organ NOC |
| X55.1 | Biopsy of lesion of unspecified organ |
| **Other Diagnostic Procedure OPCS Codes** | |
| E49.2 | ^Ϯ^Diagnostic fibreoptic endoscopic examination of lower respiratory tract and lavage of lesion of lower respiratory tract |
| E63.2 | ^Ϯ^Endobronchial ultrasound examination of mediastinum |
| E63.9 | Unspecified diagnostic endoscopic examination of mediastinum |
| Y53.2 | Approach to organ under ultrasonic control |
| Y74.4 | Thoracoscopic video-assisted approach to thoracic cavity |
| **Diagnostic Imaging OPCS Codes** | |
| U36.2 | Positron emission tomography with computed tomography NEC |
| U26.1 | Glomerular filtration rate testing |
| U05.1 | Computed tomography of head |
| U21.2 AND Y98.3 AND Z92.4 | Computed tomography NEC -Radiology of three body areas (or 20-40 minutes)- Chest NEC |
| U21.2 AND Y98.3 AND Z92.6 | Computed tomography NEC -Radiology of three body areas (or 20-40 minutes)- Abdomen NEC |
| U21.2 AND Y98.3 AND Z75.9 | Computed tomography NEC -Radiology of three body areas (or 20-40 minutes)- Bone of pelvis NEC |
| **RTDS** | |
|  | Date of Request on booking form consent date |

NEC (not elsewhere classified).NOC (not otherwise classified). ^§^Band numbers relating to the chemotherapy are assigned for costing purposes and do not help identify tumour type or origin, nor if the treatment is radical or palliative.
