# Supplementary material for: A methodology to extract outcomes from routine healthcare data for patients with locally advanced non-small cell lung cancer
Source: BMC Health Serv Res. 2018 Apr 11;18:278. doi: 10.1186/s12913-018-3029-6 (PMC5896093; doi:10.1186/s12913-018-3029-6)
Supplement: Supplementary file 3 — OPCS codes identifying primary management: Table listing radiotherapy OPCS codes, chemotherapy OPCS codes and interventional OPCS codes. (DOCX 17 kb) [file 12913_2018_3029_MOESM3_ESM.docx]

**Additional file 3. OPCS codes identifying primary management.**

| **Radiotherapy OPCS Codes** | |
| --- | --- |
| X65.4 | Delivery of a fraction of external beam radiotherapy NEC |
| Y92.1 | Technical support for preparation for radiotherapy |
| X67.1 | Preparation for intensity modulated radiation therapy |
| X67.7 | Preparation for complex conformal radiotherapy |
| Y91.1 | Megavoltage treatment for complex radiotherapy |
| Y91.4 | Megavoltage treatment for adaptive radiotherapy |
| **Chemotherapy OPCS Codes** | |
| Z51.1 | Chemotherapy session for neoplasm |
| X70.3 | ^§^Procurement of drugs for chemotherapy for neoplasm for regimens in Band 3 |
| X72.1 | Delivery of complex chemotherapy for neoplasm including prolonged infusional treatment at first attendance |
| X71.5 | ^§^Procurement of drugs for chemotherapy for neoplasm for regimens in Band 10 |
| X71.1 | ^§^Procurement of drugs for chemotherapy for neoplasm for regimens in Band 6 |
| X70.5 | ^§^Procurement of drugs for chemotherapy for neoplasm for regimens in Band 5 |
| **Interventional OPCS Codes** | |
| L76.9 | ^†^Unspecified endovascular placement of stent |
| L79.3 | ^†^Insertion of stent into vena cava NEC |

NEC (not elsewhere classified).NOC (not otherwise classified). ^§^Band numbers relating to the chemotherapy are assigned for costing purposes and do not help identify tumour type or origin, nor if the treatment is radical or palliative. ^†^These are interventional procedures undertaken for patients presenting with superior vena cava obstruction secondary to a locally advanced tumour in the apex of the lung).
