# Supplementary material for: A methodology to extract outcomes from routine healthcare data for patients with locally advanced non-small cell lung cancer
Source: BMC Health Serv Res. 2018 Apr 11;18:278. doi: 10.1186/s12913-018-3029-6 (PMC5896093; doi:10.1186/s12913-018-3029-6)
Supplement: Supplementary file 4 — ICD-10 codes indicating primary site lung malignancies: Table listing ICD-10 codes indicating primary site lung malignancy. (DOCX 17 kb) [file 12913_2018_3029_MOESM4_ESM.docx]

**Additional file 4. ICD-10 codes indicating primary site lung malignancies**

| **ICD-10 codes indicating primary site lung malignancy** | |
| --- | --- |
| C34 | Malignant neoplasm of bronchus or lung |
| C34.0 | Main bronchus, Carina, Hilum of lung |
| C34.1 | Upper lobe, bronchus or lung |
| C34.2 | Middle lobe, bronchus or lung |
| C34.3 | Lower lobe, bronchus or lung |
| C34.8 | Overlapping lesion of bronchus and lung |
| C34.9 | Malignant neoplasm of bronchus or lung, unspecified |
| C38 | Malignant neoplasm of heart, mediastinum and pleura |
| C38.3 | Malignant neoplasm of mediastinum, part unspecified |
| C38.4 | Pleura |
| C38.8 | Overlapping lesion of heart, mediastinum and pleura |
| C77.1 | *Secondary & unspecified malignant neoplasm of intrathoracic lymph nodes |
| **Additional indicator ICD-10 codes** | |
| R91 | ^§^Abnormal findings on diagnostic imaging of lung |

*This code is included in the primary presentation list of codes as patients with locally advanced NSCLC often have mediastinal lymph node disease. ^§^This code is used to denote “coin lesions not otherwise specified” and “lung mass not otherwise specified” and whilst such findings cannot confirm diagnosis, they are highly suggestive of lung malignancy and this is often the first indication to clinicians that a patient may have a lung malignancy.
