# Supplementary material for: A methodology to extract outcomes from routine healthcare data for patients with locally advanced non-small cell lung cancer
Source: BMC Health Serv Res. 2018 Apr 11;18:278. doi: 10.1186/s12913-018-3029-6 (PMC5896093; doi:10.1186/s12913-018-3029-6)
Supplement: Supplementary file 5 — ICD-10 codes indicating secondary site malignancies or complications from recurrent/ progressive/ metastatic disease: Table listing ICD-10 codes indicating secondary site malignancies and ICD-10 codes indicating complications from disease. (DOCX 18 kb) [file 12913_2018_3029_MOESM5_ESM.docx]

**Additional file 5. ICD-10 codes indicating secondary site malignancies or complications from recurrent/ progressive/ metastatic disease**

| **ICD-10 codes indicating secondary site malignancies** | |
| --- | --- |
| C77.0 | Secondary & unspecified malignant neoplasm of lymph nodes of head, face & neck |
| C77.1 | Secondary & unspecified malignant neoplasm of intrathoracic lymph nodes |
| C77.2 | Secondary & unspecified malignant neoplasm of intra-abdominal lymph nodes |
| C77.3 | Secondary & unspecified malignant neoplasm of axillary and upper limb lymph nodes |
| C77.4 | Secondary & unspecified malignant neoplasm of inguinal and lower limb lymph nodes |
| C77.5 | Secondary & unspecified malignant neoplasm of intrapelvic lymph nodes |
| C77.8 | Secondary & unspecified malignant neoplasm of lymph nodes of multiple regions |
| C78.0 | Secondary malignant neoplasm of lung |
| C78.1 | Secondary malignant neoplasm of mediastinum |
| C78.2 | Secondary malignant neoplasm of pleura; Malignant pleural effusion NOS |
| C78.3 | secondary malignant neoplasm of other and unspecified respiratory organs |
| C78.6 | Secondary malignant neoplasm of retroperitoneum & peritoneum |
| C79.0 | Secondary malignant neoplasm of kidney & renal pelvis |
| C79.2 | Secondary malignant neoplasm of skin |
| C79.3 | Secondary malignant neoplasm of brain & cerebral meninges |
| C79.5 | Secondary Malignant Neoplasm Of Bone And Bone Marrow |
| C79.7 | Secondary malignant neoplasm of adrenal gland |
| C79.8 | Secondary malignant neoplasm of other specified sites |
| C79.9 | Secondary malignant neoplasm, unspecified site; Carcinomatosis (secondary); Disseminated (secondary): cancer NOS, malignancy NOS; Generalized (secondary):cancer NOS, malignancy NOS; Multiple secondary cancer NOS |
| **ICD-10 codes indicating complications from disease** | |
| R91 | ^§^Abnormal findings on diagnostic imaging of lung |
| M50.1 | Radiculopathy-Cervical |
| G54.0 | Brachial plexus disorders |
| G55.0 | Nerve root and plexus compressions in neoplastic disease |
| G95.2 | Cord compression, unspecified |
| G83.4 | Cauda equina syndrome |

NOC (not otherwise classified). ^§^This code is used to denote “coin lesions not otherwise specified” and “lung mass not otherwise specified” and whilst such findings cannot confirm diagnosis, they are highly suggestive of lung malignancy and this is often the first indication to clinicians that a patient may have a lung malignancy.
