# Supplementary material for: A methodology to extract outcomes from routine healthcare data for patients with locally advanced non-small cell lung cancer
Source: BMC Health Serv Res. 2018 Apr 11;18:278. doi: 10.1186/s12913-018-3029-6 (PMC5896093; doi:10.1186/s12913-018-3029-6)
Supplement: Supplementary file 6 — OPCS codes identified for recurrent, progressive or metastatic disease presentation and investigation: Table listing diagnostic procedure OPCS codes, diagnostic imaging OPCS codes and RTDS indicators. (DOCX 19 kb) [file 12913_2018_3029_MOESM6_ESM.docx]

**Additional file 6. OPCS codes identified for recurrent, progressive or metastatic disease presentation and investigation**

| **Diagnostic Procedure OPCS Codes** | |
| --- | --- |
| E63.2 | Endobronchial ultrasound examination of mediastinum |
| T87.4 | Excision or biopsy of mediastinal lymph node |
| Y20.4 | Fine needle aspiration NOC |
| Y53.2 | Approach to organ under ultrasonic control |
| E59.1 | Needle biopsy of lesion of lung |
| E63.9 | Unspecified diagnostic endoscopic examination of mediastinum |
| T87.4 | Excision or biopsy of mediastinal lymph node |
| E59.3 | Biopsy of lesion of lung NEC |
| Y21.1 | Brush cytology of organ NOC |
| E49.2 | Diagnostic fibreoptic endoscopic examination of lower respiratory tract and lavage of lesion of lower respiratory tract |
| T12.3 | Aspiration of pleural cavity |
| T09.2 | Open biopsy of lesion of pleura |
| Y21.1 | Brush cytology of organ NOC |
| T12.1 | Drainage of lesion of pleura NEC |
| Y74.4 | Thoracoscopic video-assisted approach to thoracic cavity |
| X55.1 | Biopsy of lesion of unspecified organ |
| **Diagnostic Imaging OPCS Codes** | |
| U36.2 | Positron emission tomography with computed tomography NEC |
| U26.1 | Glomerular filtration rate testing |
| U21.1 AND Z06.1 | Magnetic resonance imaging NEC- Cervical spinal cord |
| U21.1 AND Z06.2 | Magnetic resonance imaging NEC- Thoracic spinal cord |
| U21.1 AND Z99.2 | Magnetic resonance imaging NEC- Intervertebral disc of thoracic spine |
| U21.1 AND Z06.3 | Magnetic resonance imaging NEC- Lumbar spinal cord |
| U05.1 | Computed tomography of head |
| U14.1 | Nuclear bone scan of whole body |
| U21.2 AND Y98.3 AND Z92.4 | Computed tomography NEC -Radiology of three body areas (or 20-40 minutes)- Chest NEC |
| U21.2 AND Y98.3 AND Z92.6 | Computed tomography NEC -Radiology of three body areas (or 20-40 minutes)- Abdomen NEC |
| U21.2 AND Y98.3 AND Z75.9 | Computed tomography NEC -Radiology of three body areas (or 20-40 minutes)- Bone of pelvis NEC |
| **RTDS** | |
|  | Date of Request on booking form consent date |

NEC (not elsewhere classified). NOC (not otherwise classified).  ^§^Band numbers relating to the chemotherapy are assigned for costing purposes and do not help identify tumour type or origin, nor if the treatment is radical or palliative. *Whilst “Preparation for intensity modulated radiation therapy” implies complex radiotherapy that is usually delivered in the radical setting, it is also used to code for SABR (stereotactic ablative radiotherapy), which can be a used for oligometastatic (single or few systemic metastases that are amenable to surgery or ablative therapy) disease.
