# Supplementary material for: A methodology to extract outcomes from routine healthcare data for patients with locally advanced non-small cell lung cancer
Source: BMC Health Serv Res. 2018 Apr 11;18:278. doi: 10.1186/s12913-018-3029-6 (PMC5896093; doi:10.1186/s12913-018-3029-6)
Supplement: Supplementary file 7 — OPCS codes identifying secondary management: Table listing radiotherapy OPCS codes, chemotherapy OPCS codes and interventional OPC codes. (DOCX 18 kb) [file 12913_2018_3029_MOESM7_ESM.docx]

**Additional file 7. OPCS codes identifying secondary management.**

| **Radiotherapy OPCS Codes** | |
| --- | --- |
| X65.4 | Delivery of a fraction of external beam radiotherapy NEC |
| X67.5 | Preparation for simple radiotherapy with imaging and simple calculation |
| Y91.2 | Megavoltage treatment for simple radiotherapy |
| X67.1 | Preparation for intensity modulated radiation therapy * |
| E59.5 | Percutaneous radiofrequency ablation of lesion of lung |
| **Chemotherapy OPCS Codes** | |
| Z51.1 | Chemotherapy session for neoplasm |
| X70.3 | ^§^Procurement of drugs for chemotherapy for neoplasm for regimens in Band 3 |
| X72.1 | Delivery of complex chemotherapy for neoplasm including prolonged infusional treatment at first attendance |
| X71.5 | ^§^Procurement of drugs for chemotherapy for neoplasm for regimens in Band 10 |
| X73.1 | Delivery of exclusively oral chemotherapy for neoplasm |
| X71.1 | ^§^Procurement of drugs for chemotherapy for neoplasm for regimens in Band 6 |
| **Interventional OPC Codes** | |
| L79.3 | ^†^Insertion of stent into vena cava NEC |
| L76.9 | ^†^Unspecified endovascular placement of stent |
| T10.2 | Endoscopic pleurodesis using talc |

NEC (not elsewhere classified).*Whilst “Preparation for intensity modulated radiation therapy” implies complex radiotherapy that is usually delivered in the radical setting, it is also used to code for SABR (stereotactic ablative radiotherapy), which can be a used for oligometastatic (single or few systemic metastases that are amenable to surgery or ablative therapy) disease. ^§^Band numbers relating to the chemotherapy are assigned for costing purposes and do not help identify tumour type or origin, nor if the treatment is radical or palliative. ^†^These are interventional procedures undertaken for patients presenting with superior vena cava obstruction secondary to a locally advanced tumour in the apex of the lung).
