# Supplementary material for: A methodology to extract outcomes from routine healthcare data for patients with locally advanced non-small cell lung cancer
Source: BMC Health Serv Res. 2018 Apr 11;18:278. doi: 10.1186/s12913-018-3029-6 (PMC5896093; doi:10.1186/s12913-018-3029-6)
Supplement: Supplementary file 8 — ECOG/ WHO Performance Status: Table describing ECOG/ WHO Performance status grades used to reflecting patients’ fitness. (DOCX 15 kb) [file 12913_2018_3029_MOESM8_ESM.docx]

**Additional file 8. ECOG/ WHO Performance Status** [17]

| **Score** |  |
| --- | --- |
| 0 | Fully active, able to carry on all pre-disease performance without restriction |
| 1 | Restricted in physically strenuous activity, but ambulatory and able to carry out work of a light and sedentary nature |
| 2 | Ambulatory and capable of all self-care but unable to carry out any work activities. Up and about more than 50% of waking hours |
| 3 | Capable of only limited self-care, confined to bed or chair more than 50% of waking hours |
| 4 | Completely disabled. Cannot carry on any self-care. Totally confined to bed or chair |
| 5 | Dead |

ECOG/ WHO Performance status is a graded score reflecting patients’ fitness. It is used by clinicians to guide decisions on patients’ suitability for intensive radical treatment and their prognosis. Patients with PS 0-1 are considered fit for radical treatment. There may be exceptional cases where patients with PS2 are offered a radical dose of radiotherapy alone but this is not standard practice and should be done with caution. It would not be appropriate to offer radical treatment to patients with a PS of 3-4 and best supportive care is the mainstay treatment for those patients[19].
